# SiCST1, a novel plant-specific protein of foxtail millet, confers cold stress tolerance in plants

**DOI:** 10.3389/fpls.2025.1618053

**Published:** 2025-08-05

**Authors:** Fan Yang, Jiaqi Qiao, Xiao Zhang, Zhuoya Zhang, Dongao Huo

**Affiliations:** ^1^ College of Biological Sciences and Technology, Taiyuan Normal University, Taiyuan, China; ^2^ Modern Agricultural Development Center, Jincheng Agricultural and Rural Bureau, Jincheng, China

**Keywords:** foxtail millet, cold stress, SiCST1, SiOFP1, BR

## Abstract

Foxtail millet (*Setaria italica*) is a significant cereal crop, but its yield is limited by extreme temperature, particularly cold stress. In this study, we identified a novel plant-specific gene, *SiCST1* (Cold Stress Tolerance in *Setaria italica* 1) in foxtail millet, through transcriptome deep sequencing (RNA-Seq) of cold-stressed seedlings. We generated a CRISPR/Cas9-mediated knockout mutant of rice homolog of *SiCST1* (designated *oscst1*). Compared to wild-type rice, *oscst1* mutant seedlings exhibited cold sensitivity with a 46% survival rate reduction under cold stress. This impaired cold stress tolerance was rescued by complementation with *SiCST1*, indicating the vital role of *SiCST1* in cold stress tolerance. *SiCST1* consists of a single exon and contains a predicted ribonuclease H-like domain. Further analysis revealed that *SiCST1* was significantly up-regulated in response to cold stress and was localized in nucleus. Additionally, our findings suggested that SiCST1 interacted with the OVATE family protein SiOFP1. The lamina joint bending assays were employed to investigate whether mutation of rice homolog *of SiCST1* affected the brassinolide (BR) signaling pathways. It was found that *oscst1* exhibited insensitivity to exogenous BR treatment. We propose a regulatory mechanism in which SiCST1 interacted with SiOFP1 to release its inhibition of BR signaling transcription complex, thereby activating BR signaling pathways and conferring cold stress tolerance. Our study provides evidence that SiCST1 is a novel plant-specific protein with an essential function involved in cold stress resistance in foxtail millet.

## 1 Introduction

Foxtail millet (*Setaria italica* (L.) Beauv.), a cultivated species within the genus *Setaria* in the *Poaceae* family, originated in China approximately 8700 years ago, having been domesticated from its wild relative, *Setaria viridis* (Bettinger et al., 2010). Foxtail millet is a significant crop and a staple component of human diets, ranking second in the global millet production (Anonymous, 2023). The completion of the whole-genome sequencing of foxtail millet revealed a relatively small genome size (approximately 490 Mb) with a low DNA repetition rate (24%) (Bennetzen et al., 2012). Furthermore, compared to its ancestral grass lineages, the genome structure of foxtail millet is highly conserved, making it an ideal model species for genetic and molecular studies (Devos et al., 1998; Jayaraman et al., 2008; Bennetzen et al., 2012). Additionally, due to its close phylogenetic relationship with important biofuel crops such as switchgrass, napiergrass, and pearl millet, foxtail millet is also regarded as a suitable model for research on these crops (Doust et al., 2009). During its growth cycle, foxtail millet is highly sensitive to cold stress, particularly during the seedling and booting stages, where cold stress can significantly hinder growth and reduce final yield (Lyons, 1973; Thakur et al., 2010; Liu et al., 2020; Zhang et al., 2022; Huang et al., 2023b). Cold stress tolerance in foxtail millet is a crucial determinant of its growing season length and geographical distribution. Therefore, enhancing its cold stress tolerance has become a primary objective in breeding programs. To achieve this goal, it is particularly vital to profoundly investigate the regulatory mechanisms of cold signaling pathways in foxtail millet.

An adaptive response called cold acclimation has evolved in temperate plants to enhance their cold stress tolerance (Thomashow, 1999; Ding and Yang, 2022). Cold acclimation in *Arabidopsis* is primarily mediated by three *CBF*/*DREB1* genes that play central and redundant roles (Novillo et al., 2007; Jia et al., 2016; Zhao et al., 2016; Wang et al., 2023a). Under cold stress, *CBF* genes are rapidly and highly induced, and the proteins they encode activate the expression of *COR* (*COLD REGULATED*) genes. This lead to the accumulation of protective substances such as osmolytes and cold-protective proteins, ultimately facilitating cold acclimation and increasing cold stress tolerance (Thomashow, 1999; Shi et al., 2018; Ding et al., 2020). The regulation of *CBF* gene expression involves various transcription factors. ICE1 and its homologous protein ICE2 positively regulate the expression of *CBFs* and cold stress tolerance (Chinnusamy et al., 2003; Fursova et al., 2009; Tang et al., 2020; Wang et al., 2023b). The circadian clock is closely associated with the cold stress response (Lu et al., 2020; Poikela et al., 2021). The rhythmic expression of *CBFs* is regulated by core components of the circadian clock, including CCA1, LHY, and PRRs (Kidokoro et al., 2021). The cold signaling pathways are also modulated by light and photoperiod (Franklin and Whitelam, 2007; Jiang et al., 2017; Wang et al., 2019). Plant hormones also play roles in plant response to cold stress, including BR (brassinosteroid), ethylene, and JA (jasmonic acid), among others (Hu et al., 2013; Huang et al., 2023a; He et al., 2024; Wang et al., 2024a, b; Zeng et al., 2025).

BR signaling constitutes a pivotal pathway in the acquisition of cold stress tolerance by plants. During cold stress, an elevation in endogenous BR content was observed in plants. When tomato (*Solanum lycopersicum* L.) leaves were exposed to 8°C for 8 h, three detectable BRs (brassinolide (BL), castorosterone (CS), and 28-norCS) were found to exhibit increased levels (Fang et al., 2019). Investigation into receptor kinases and transcription factors involved in BR signaling have demonstrated that the overexpression of positive components of BR signaling enhances cold stress tolerance, whereas the overexpression of negative components impairs it. In *Arabidopsis*, the overexpression of *BRI1* markedly improves plant survival under cold stress condition, whereas the survival of *bri1–1* and *bri1–301* mutant plants was significantly compromised (Eremina et al., 2016). BIN2 functions as a negative component within BR signaling pathways. Notably, triple mutant plants of *BIN2* exhibit enhanced stress cold resistance, whereas the overexpression of *BIN2* in plants leads to decreased cold stress resistance (Li et al., 2017). Studies have further indicated that the exogenous application of BR increases the expression of *COR* genes and thereby enhances cold stress tolerance (Kagale et al., 2007).

In our study, based on RNA-Seq analysis of cold-stressed foxtail millet seedlings, *SiCST1* (*LOC101781117*) was selected as one of the most significantly up-regulated genes for functional validation. This study demonstrated its role as a key regulator of cold stress tolerance and revealed the underlying molecular mechanism. The discovery of the interaction between SiCST1 and SiOFP1, as well as its implication for BR signaling and cold stress tolerance, offers new avenues for research into genetic and molecular basis of crop stress resilience. The findings have the potential to facilitate the rapid development of new varieties with enhanced cold stress tolerance.

## 2 Materials and methods

### 2.1 Plant material

Plants were grown under natural condition in Jinzhong (36°85’ N, 111°77’ E), Shanxi province, China. Yugu1 (*Setaria italica* cv. Yugu1), a sequenced wild-type foxtail millet variety with a complete reference genome (Bennetzen et al., 2012), was employed in this study.

The pYLCRISPR was used to construct CRISPR vector targeting *OsCST1* (rice homolog *of SiCST1*), which were subsequently employed for genetic transformation of rice variety Zhonghua11 (*Oryza sativa* L. ssp. *japonica* Zhonghua11). CRISPR-P 2.0 was utilized to design the base-pairing sequence of the sgRNA (5′-GCATCAGCAGCAGACGCCAC-3′) targeting the single exon of *OsCST1*.

To complement *oscst1* mutant, genomic fragments of *SiCST1* (3609 bp) were cloned into pCAMBIA1301 vector. The resulting construct was transformed into *Agrobacterium tumefaciens* EHA105 and subsequently transformed into rice callus induced from mature seeds of *oscst1* mutant.

### 2.2 Cold stress treatment

Cold stress treatment were implemented as previously described (Ma et al., 2009). The rice seedlings were cultivated in Kimura B nutrient solution under controlled condition (day/night temperature: 30°C/25°C and light/dark photoperiod: 10 h/14 h) until they reached the three-leaf stage. Subsequently, the seedlings were subjected to cold stress (4°C) for 96 h. After a recovery period of 7 days at 30°C/25°C (day/night), seedling survival rate were assessed. Three biological replicates were established, each comprising 32 rice seedlings.

### 2.3 RNA-seq and gene expression analysis

For RNA-Seq analysis under cold stress, three-leaf-stage foxtail millet seedlings grown at 28°C/24°C (day/night) were transferred to 4°C. After 24 h of treatment, both cold-treated and untreated seedlings were harvested, immediately frozen in liquid nitrogen, and stored at -80°C for subsequent RNA isolation. Total RNA was extracted from three biological replicates (each comprising five pooled seedlings). High-throughput sequencing was performed on HiSeq 2500 (Novogene, Beijing). Clean reads were aligned to *Setaria italica* reference genome (v2.2, Phytozome).

For *SiCST1* expression analysis during cold stress treatment, foxtail millet seedlings cultivated to the three-leaf stage at 28°C/24°C (day/night) were exposed to 4°C. The seedlings were collected at 0, 1, 3, 6, 12, and 24 h post-treatment, immediately frozen in liquid nitrogen, and stored at -80°C for RNA extraction. Total RNA was extracted from three biological replicates, with each consisting of a pooled sample of five seedlings.

Total RNA was extracted from tissues using the RNeasy plant mini kit (QIAGEN). The isolated RNA was reverse transcribed using SuperScript III reverse transcriptase (Invitrogen). Quantitative RT-PCR (RT-qPCR) was performed utilizing SYBR Green Real-Time PCR Master Mixes (Invitrogen). Gene expression level was normalized with foxtail millet *actin* gene (*Seita.7G294000*). Primers used for RT-qPCR are listed in **Supplementary Table S1**.

### 2.4 Subcellular localization

The full-length cDNA of *SiCST1* was cloned into pBI221-GFP vector to create fusion protein with GFP at the C-terminus of SiCST1. SiCST1-GFP and the nucleus marker H2B-mCherry were co-transformed into foxtail millet protoplasts via the polyethylene glycol-mediated transformation method, as previously described (Chen et al., 2006). After culturing at 25°C for 16 h in darkness, the transformed protoplasts were observed using a confocal scanning microscope.

### 2.5 Yeast two-hybrid analysis

For bait construction, the full-length *SiCST1* cDNA was cloned into pGBKT7 (Clontech) and transformed into yeast strain Y2HGold. For prey cDNA library construction, total RNA was isolated from 2-week-old foxtail millet seedlings (Yugu1) using the RNeasy Plant Kit (QIAGEN). Synthesized cDNA was co-transformed with linearized pGADT7-Rec vector (Clontech) into *Saccharomyces cerevisiae* strain Y187 via the Make Your Own Mate & Plate Library System (Clontech).

For Y2H screening, bait strain Y2HGold (harboring pGBKT7-SiCST1) and prey strain Y187 (containing the cDNA library) were mated in YPDA liquid medium at 30°C for 24 h with 40 rpm shaking. Mating diploids were then plated onto SD/-Leu/-Trp/-His/-Ade + X-α-Gal plates. Following 7-day incubation at 30°C, colonies exhibiting blue coloration were restreaked for confirmation. Validated positive clones underwent PCR amplification of insert fragments, followed by DNA sequencing. Sequence alignment against *Setaria italica* genome using BLASTn identified SiOFP1 (LOC101755245) as a high-confidence interactor detected in 12 out of 20 sequenced clones.

### 2.6 Bimolecular fluorescence complementation analysis

The full-length cDNA of *SiCST1* was cloned into pUC-SPYNE173 vector and the full-length cDNA of *SiOFP1* was cloned into pUC-SPYCE (M) vector. To evaluate potential false positives, the pUC-SPYNE173-*SiCST1* construct was co-transformed with the empty pUC-SPYCE (M) vector as a negative control.

Protoplasts were isolated from 10-day-old etiolated foxtail millet leaves (Yugu1) by enzymatic digestion. Leaves were sliced into 0.5-mm strips and incubated in digestion solution [1.5% (w/v) Cellulase R10, 0.4% (w/v) Macerozyme R10, 0.5 M D-mannitol, 20 mM KCl, 20 mM MES (pH 5.7), 10 mM CaCl_2_, 0.1% BSA] at 28°C for 4 h with gentle shaking (40 rpm). Protoplasts were filtered through 75-μm nylon mesh, washed twice with W5 solution [154 mM NaCl, 125 mM CaCl_2_, 5 mM KCl, 2 mM MES (pH 5.7)], and resuspended in MMg solution [0.5 M mannitol, 15 mM MgCl_2_, 4 mM MES (pH 5.7)] at a density of 1 - 5 × 10^6^ cells/mL.

For transformation, 10 μg each of pUC-SPYNE173-*SiCST1* (N-terminal YFP fragment) and pUC-SPYCE(M)-*SiOFP1* (C-terminal YFP fragment) plasmids were added to 100 μL protoplast suspension, followed by 110 μL PEG solution (40% PEG4000, 0.4 M mannitol, 0.1 M CaCl_2_). After 15-min incubation at 25°C, reactions were quenched with 1 mL W5 solution. Transfected protoplasts were cultured in WI solution [0.5 M D-mannitol, 4 mM MES (pH 5.7), 20 mM KCl] at 25°C for 16 h in darkness (Walter et al., 2004). Following incubation, YFP fluorescence was observed using a confocal scanning microscope.

### 2.7 Co-immunoprecipitation

The pA7-SiOFP1-GFP and FLAG-SiCST1 plasmids were co-transformed into foxtail millet protoplasts with FLAG-SiCST1 and the empty pA7-GFP vector serving as control to assess false positives. Transfected protoplasts were incubated at 25°C in darkness for 14–16 h. Upon microscopic confirmation of GFP fluorescence expression, the protoplasts were lysed with 400 μL IP buffer (10 mM HEPES, 100 mM NaCl, 1 mM EDTA, 10% glycerol, 0.5% Triton X-100, 1×protease inhibitor cocktail, pH 7.5). The lysate was centrifuged at 5,000 g for 5 min at 4°C. The resultant supernatant was incubated overnight at 4°C with 20 μL of anti-FLAG agarose beads (MBL). The beads were collected and washed five times with IP buffer, followed by boiling in SDS buffer. The samples were examined by western blotting using anti-GFP or anti-FLAG antibody.

### 2.8 Lamina joint bending assays

Lamina joint bending assays were performed on the second fully expanded leaves of two-week-old WT and *oscst1* rice seedlings as previously described (Feng et al., 2016). Excised leaf segments were placed in media supplemented with 0, 0.01, 0.1, or 1 μM 24-epibrassinolide, with three biological replicates per concentration and five seedlings per replicate. After 48 h incubation in darkness at 28°C, the lamina joint angle was quantified using ImageJ software.

### 2.9 Statistical analysis

Significant differences between control and treatment were analyzed using Student’s *t*-test within SPSS version 25 software (IBM SPSS Statistics). Differences were considered significant at *P* < 0.05 and highly significant at *P* < 0.01.

## 3 Results

### 3.1 SiCST1 exhibits the ability to confer cold stress tolerance

To identify genes associated with cold stress tolerance in foxtail millet at the seedling stage, we conducted transcriptome deep sequencing (RNA-Seq) analysis on three-leaf-stage seedlings of Yugu1, which were exposed to 4°C cold stress for 24 h. We thus identified *SiCST1* (*LOC101781117*) was up-regulated 4-fold by cold stress treatment. To explore the possible involvement of *SiCST1* in cold stress tolerance, we developed CRISPR/Cas9 knockout mutant line, *oscst1*, targeting the homolog of *SiCST1* in rice (*OsCST1*, *LOC4329262*). *OsCST1* was identified by conducting a blastp search of SiCST1 protein sequence against rice protein database (*Oryza sativa* ssp. *japonica*). Genomic analysis confirmed that *OsCST1* is a single-copy gene in rice. Knockout of *OsCST1* yielded *oscst1* mutant. Sequencing of PCR products from *oscst1* T1 generation plants confirmed a 1-bp insertion in the single exon of *OsCST1*, causing a frameshift and premature termination (**Supplementary Figures S1A–C**). The truncated protein consisting of 405 amino acids lacks the C-terminal domain (**Supplementary Figure S1D**).

**Figure 1:**
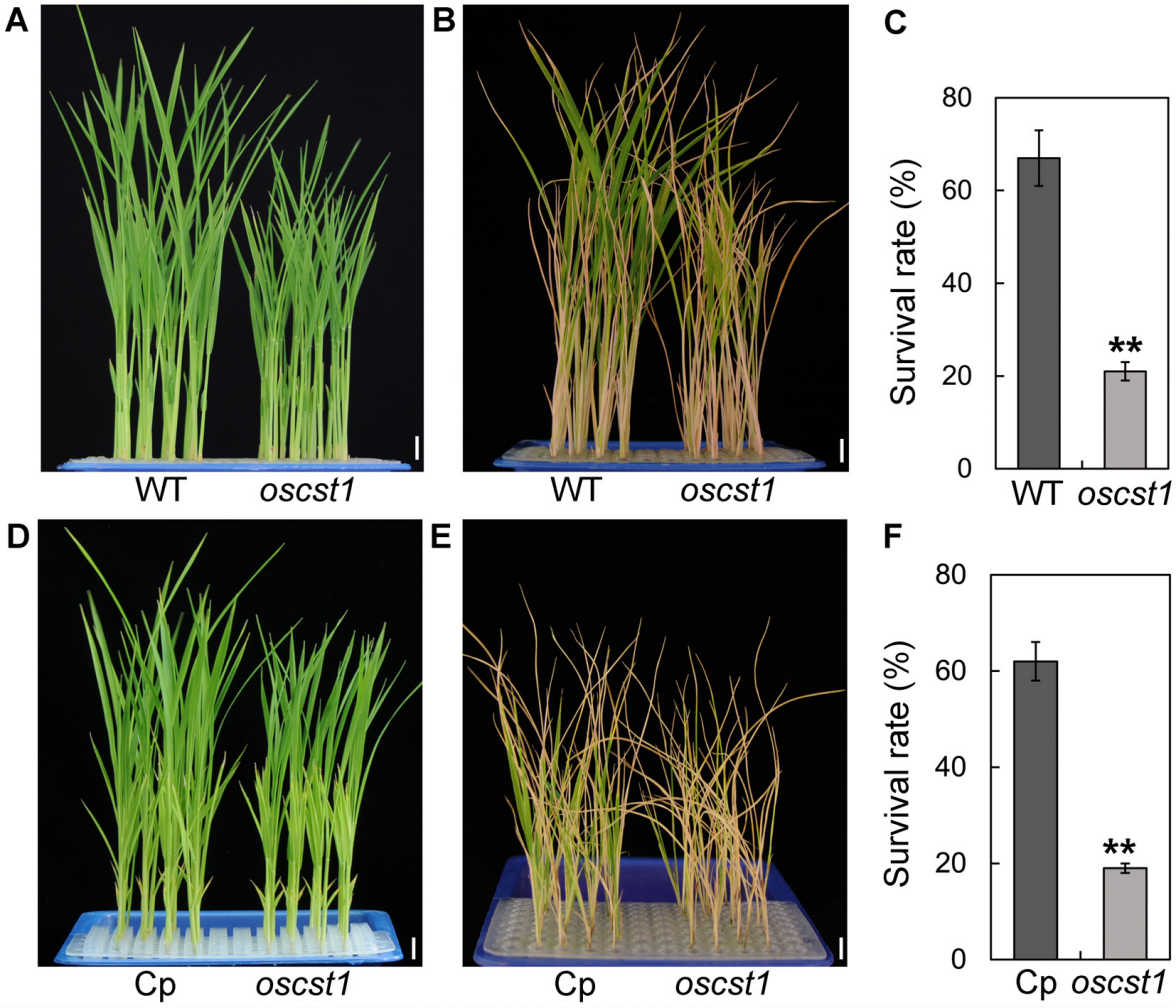
*SiCST1* is essential for cold stress tolerance. **(A, D)** Nontreatment control. **(B, E)** Cold stress treatment. **(C, F)** Survival rate was assessed following cold stress treatment at 4°C for 96 h and subsequent recovery at 30°C for 7 days. WT, wild-type; Cp, complementary line. Scale bar, 1 cm. Student’s *t*-test, **P < 0.01.

We then proceeded to evaluate the cold stress tolerance of T1 generation plants of *oscst1* by exposing seedlings to 4°C cold stress followed by recovery at 30°C. We defined plants possessing cold stress tolerance as those that displayed continued leaf growth or newly differentiated leaves upon being returned to normal condition following cold stress treatment. A significant difference in survival rate (percentage of seedlings that survived the treatment) and seedling height was observed between wild-type (WT) and *oscst1* mutant (**Figures 1A–C**; **Supplementary Figure S2**). Notably, *oscst1* mutant seedlings demonstrated cold sensitivity compared to WT. Furthermore, we genetically transformed *oscst1* with FLAG-tagged genomic fragments of *SiCST1*. The resultant complementary line (Cp) rescued the impaired cold stress tolerance observed in *oscst1* (**Figures 1D–F**; **Supplementary Figure S2**). Taken together, these findings imply a pivotal regulatory role for *SiCST1* in modulating cold stress tolerance.

### 3.2 SiCST1 is a plant‐specific protein with a ribonuclease H‐like domain

**Figure 2:**
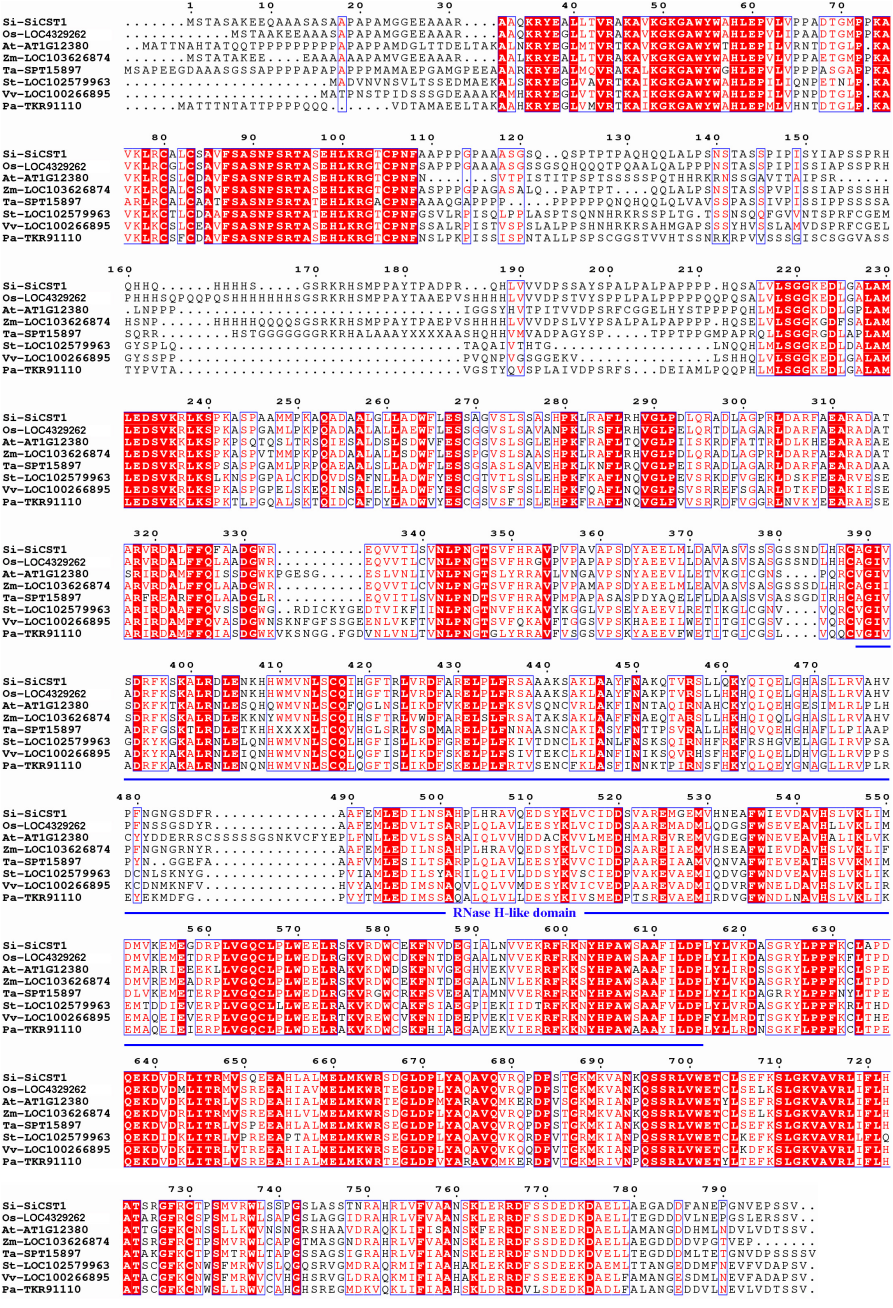
Multiple sequence alignments were performed using MEGA software. These homolog sequences used are LOC4329262 from *Oryza sativa* (Os), At1g12380 from *Arabidopsis thaliana* (At), LOC103626874 from *Zea mays* (Zm), SPT15897 from *Triticum aestivum* (Ta), LOC102579963 from *Solanum tuberosum* (St), LOC100266895 from *Vitis vinifera* (Vv) and TKR91110 from *Polulus alba* (Pa). The RNase H‐like domain was marked with a blue underline.

**Figure 3:**
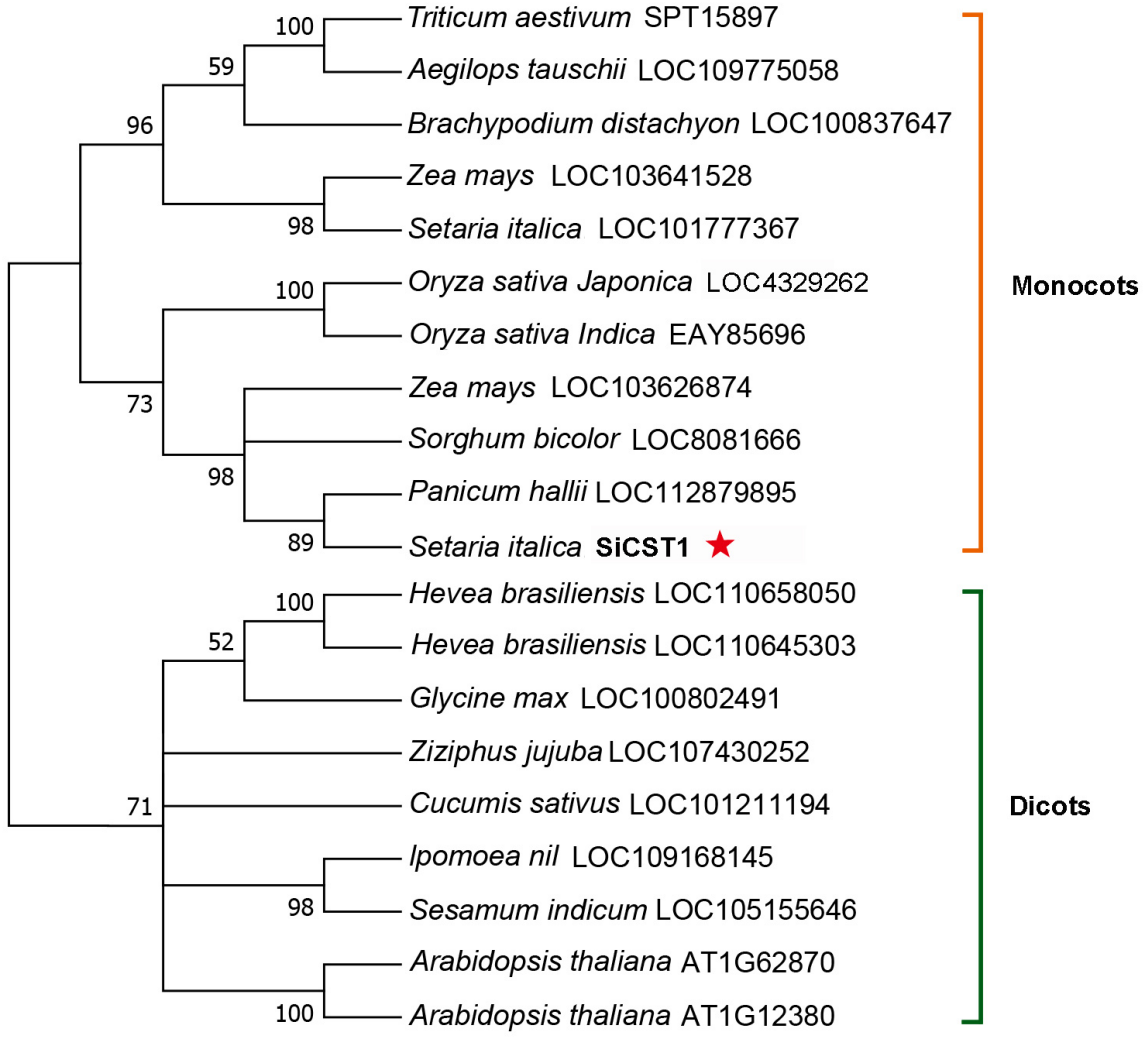
Phylogenetic tree of SiCST1 and its homologs across different species. The construction of the phylogenetic tree was accomplished utilizing MEGA11 software, employing the maximum-likelihood method with 1000 bootstrap replicates.

A full-length *SiCST1* cDNA was synthesized using total RNA extracted from two-week-old seedlings of foxtail millet (Yugu1). Sequence analysis revealed that SiCST1 consists of a single exon and has an open reading frame (ORF) encoding 798 amino acid residues, with predicated molecular weight of 87032.1 and isoelectric point of 8.6. Domain analysis revealed that SiCST1 possesses a ribonuclease H‐like (RNase H‐like) domain, which was conserved in SiCST1 and its homologs (**Figure 2**). Sequence homology searches indicated the presence of SiCST1 homologs in various spermatophytes, but notably absent in animals. Phylogenetic analysis distinctly categorized these sequences into separate clades corresponding to monocots and dicots (**Figure 3**), suggesting that the emergence of these sequences may be associated with the differentiation of spermatophytes.

**Figure 4:**
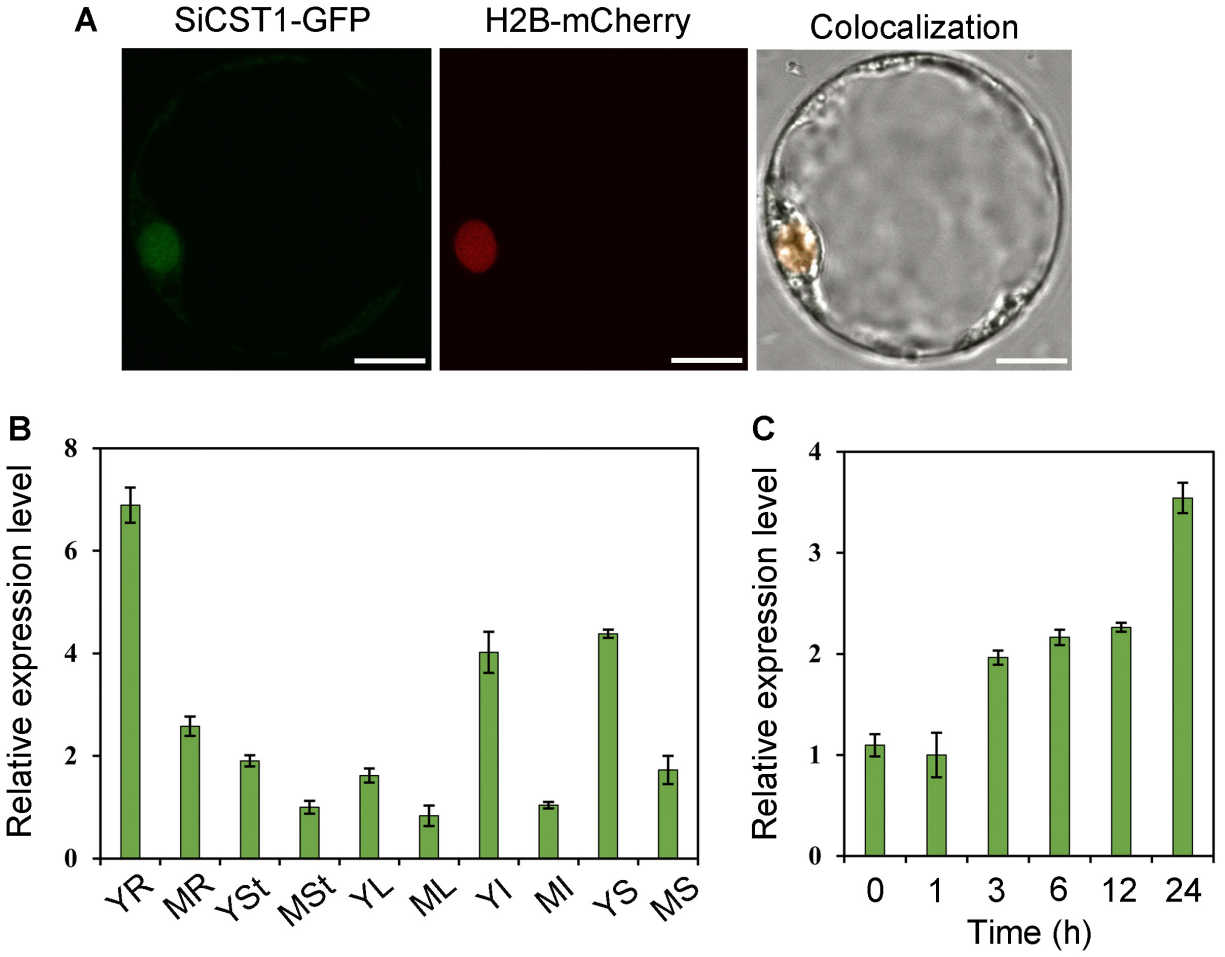
Subcellular localization and expression pattern of SiCST1. **(A)** Nucleus localization of SiCST1 in foxtail millet protoplasts. Scale bar, 5 μm. **(B)** Tissue-specific expression of *SiCST1*. Total RNA was extracted from young roots (YR), mature roots (MR), young stems (YSt), mature stems (MSt), young leaves (YL), mature leaves (ML), young inflorescences (YI), mature inflorescences (MI), young seeds (YS), and mature seeds (MS), respectively. **(C)** Accumulation of *SiCST1* transcripts in response to cold stress treatment. Values represented means ± SE (n = 15).

To identify the subcellular localization of SiCST1, we performed transient protoplast transformation of foxtail millet using constructs of either *ubi*::SiCST1-GFP (maize ubiquitin promoter-driven fusion) or H2B-mCherry (constituting a nucleus marker). We observed complete overlap between SiCST1-GFP and H2B-mCherry fluorescence signals (**Figure 4A**), indicating that SiCST1 is localized in nucleus. Constitutive expression of *SiCST1* was observed in all examined tissues, with particularly high expression level detected in young tissues (**Figure 4B**). Additionally, exposure to cold stress (4°C) induced *SiCST1* expression, with expression level more than three times higher after 24 h of treatment compared to the untreated control (**Figure 4C**). This discovery was consistent with the involvement of this gene in seedling cold stress tolerance.

### 3.3 SiCST1 interacts with SiOFP1

**Figure 5:**
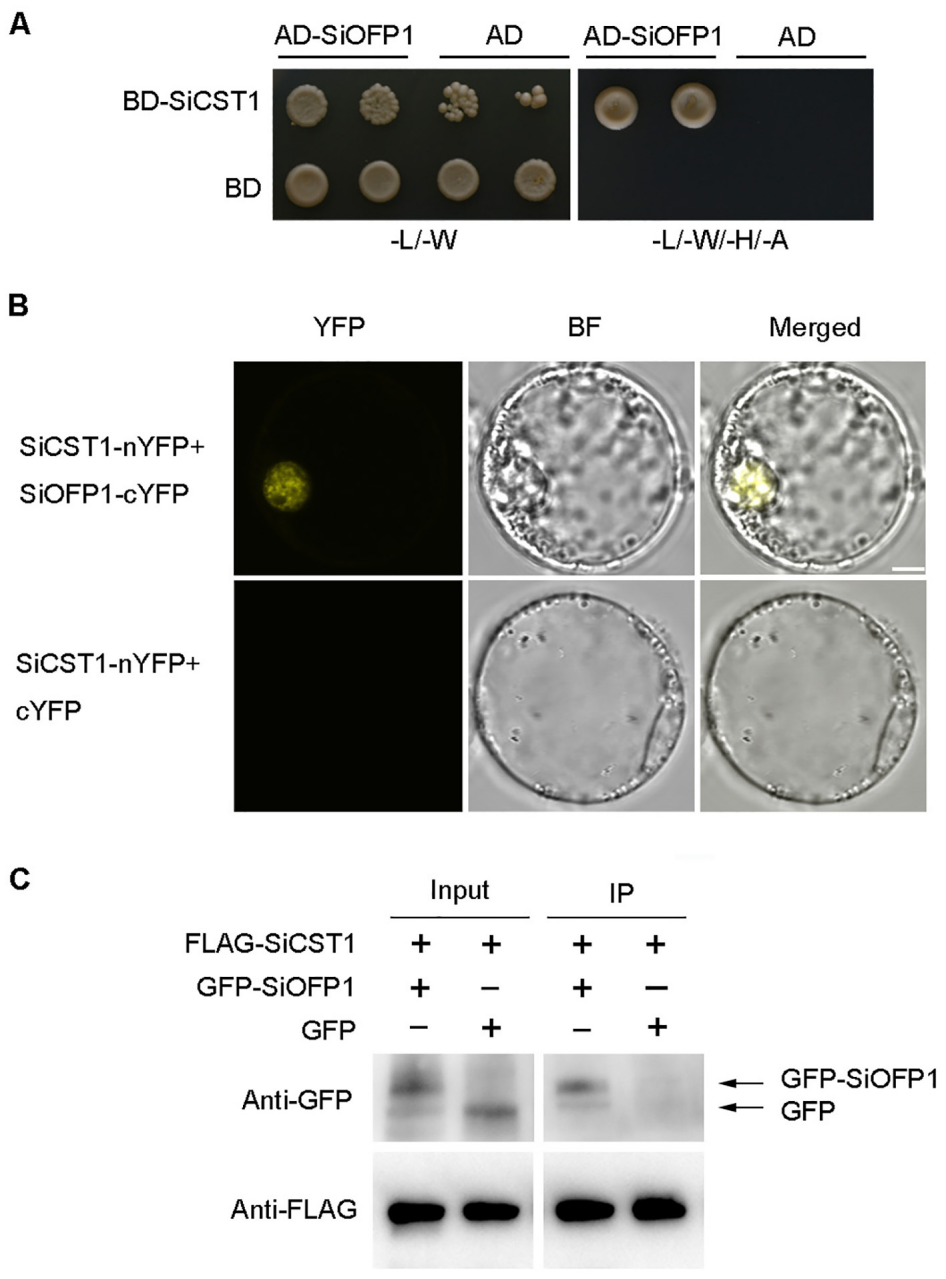
SiCST1 interacts with SiOFP1. **(A)** Interaction between SiCST1 and SiOFP1 in Y2H assay. **(B)** BiFC in foxtail millet protoplasts confirmed the interaction between SiCST1 and SiOFP1. Scale bar, 5 μm. **(C)** CoIP analysis further verified the interaction between SiCST1 and SiOFP1. FLAG-SiCST1 was precipitated from transfected protoplast lysates using anti-FLAG agarose beads, and the interaction was detected by western blotting with anti-GFP or anti-FLAG antibody.

To elucidate how SiCST1 regulates cold stress tolerance, potential SiCST1-interacting proteins were identified using prey cDNA library generated from foxtail millet seedling RNA and a bait expressing the full-length cDNA of *SiCST1*. The yeast two-hybrid system (Y2H) revealed a potential interaction between SiCST1 and SiOFP1 (LOC101755245) (**Figure 5A**). The OFPs are a group of OVATE family transcription factors characterized by a conserved OVATE domain. This interaction was further verified via bimolecular fluorescence complementation (BiFC) and co-immunoprecipitation (CoIP) assays. In BiFC assay, SiCST1 and SiOFP1 were fused to the N- and C-termini of YFP, respectively, generating SiCST1-nYFP and SiOFP1-cYFP constructs. Co-expression of these constructs in foxtail millet protoplasts resulted in fluorescence signals, confirming the interaction between SiCST1 and SiOFP1 through YFP reconstitution. Conversely, protoplasts co-expressing SiCST1-nYFP and the control construct cYFP failed to generate any fluorescence signals (**Figure 5B**). In CoIP assay, co-expression of FLAG-SiCST1 and GFP-SiOFP1 in foxtail millet protoplasts revealed co-precipitation of FLAG-SiCST1 and GFP-SiOFP1, confirming that SiCST1 directly interacts with SiOFP1 *in vivo* (**Figure 5C**).

### 3.4 SiCST1 was involved in BR signaling pathways

**Figure 6:**
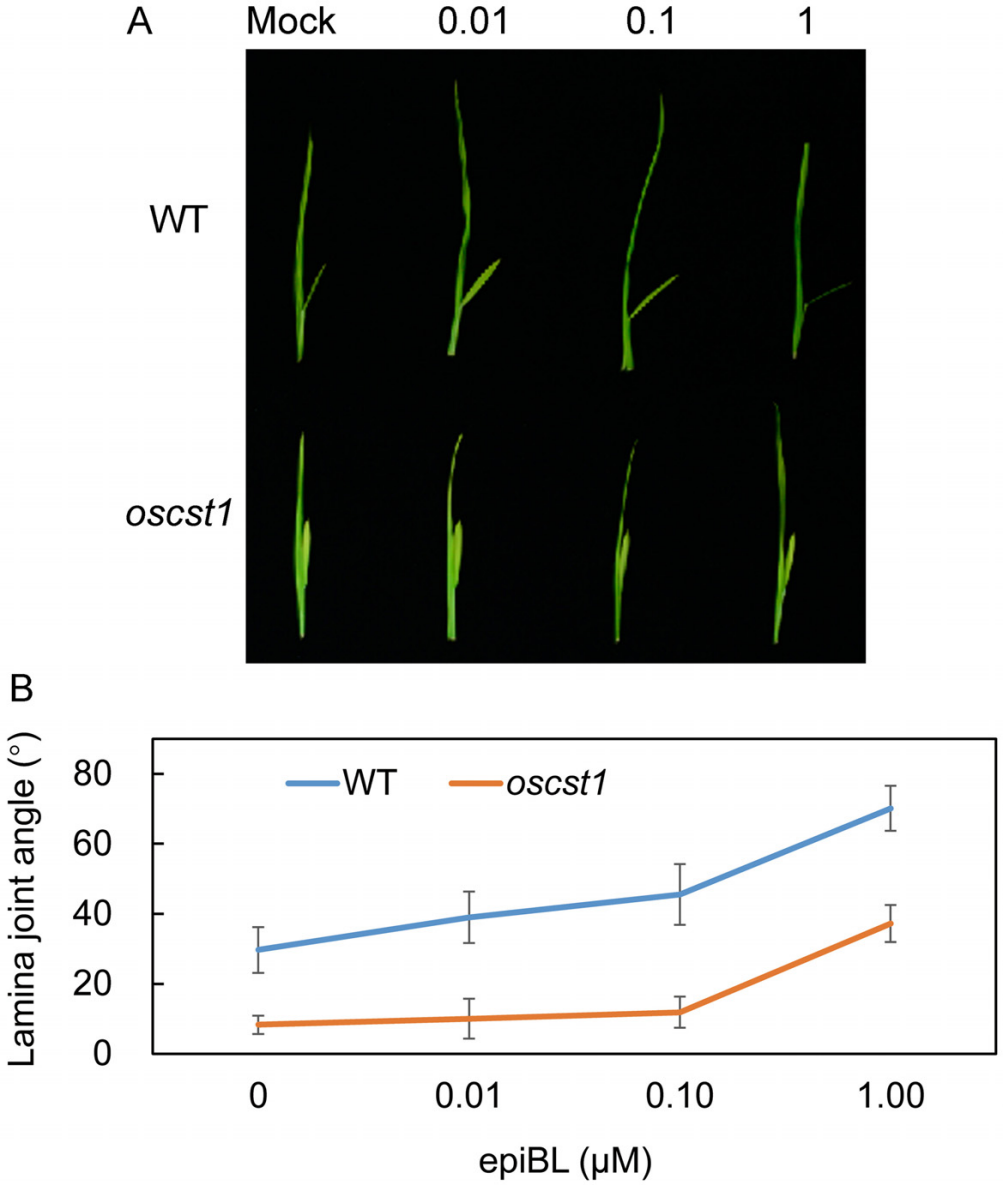
*oscst1* exhibited reduced sensitivity to BR. **(A)** The lamina joint bending response was assessed in WT and *oscst1* following application of various concentrations (μM) of epiBL, utilizing the excised leaf segment method. **(B)** Quantification of lamina joint angles shown in **(A)**. Values represented means ± SD (n = 15).

Several studies have reported that OFPs play regulatory roles in phytohormone signaling and biosynthesis pathways, particularly in BR signaling (Snouffer et al., 2020), leading us to formulate the speculation that *SiCST1* might be implicated in BR signaling pathways. To rigorously test this hypothesis, a series of lamina joint bending assays were conducted on both *oscst1* mutant and WT. The results revealed that in WT, the lamina joint angle gradually increased with rising concentrations of 24-epibrassinolide (epiBL). In contrast, *oscst1* mutant demonstrated reduced sensitivity to exogenous BR treatment, even at high concentrations of epiBL (**Figures 6A, B**). These observations provide preliminary evidence supporting the involvement of *SiCST1* in BR signaling pathway.

## 4 Discussion

### 4.1 Identification and function of SiCST1 in cold stress tolerance

The exploration of cold stress tolerance in foxtail millet has become a central focus in agricultural research, driven by the urgent need to enhance crop resilience in the face of global climate change. To date, most research efforts concentrated on elucidating the physiological changes, transcriptomic response, and metabolic adaptations that occur in foxtail millet following cold stress exposure (Ananthi et al., 2023; Zhang et al., 2023; Zhao et al., 2023). These studies provided invaluable insights into the multifaceted nature of foxtail millet’s cold tolerance mechanisms. Physiological studies demonstrated that foxtail millet underwent a series of adaptive physiological changes in response to cold stress (Ananthi et al., 2023). Transcriptomic analysis further complemented these findings by revealing a complex network of genes that were either up- or down-regulated in response to cold stress, hinting at the intricate regulatory pathways involved in cold stress tolerance (Zhang et al., 2023). Additionally, metabolic profiling studies identified specific metabolites that accumulated or decreased under cold stress, offering clues into the metabolic reprogramming that occurred in foxtail millet to cope with cold stress (Zhao et al., 2023).

While previous studies have significantly expanded our understanding of foxtail millet cold stress tolerance, a critical gap persists in our knowledge of the underlying molecular mechanisms. This limitation hinders the development of targeted genetic engineering or breeding strategies to enhance cold tolerance. In this context, the identification of *SiCST1* as a novel plant-specific gene with a predicted ribonuclease H-like domain in foxtail millet represents a significant step forward in our understanding of cold stress tolerance mechanisms. Our results demonstrated that *SiCST1* played a crucial role in conferring cold stress tolerance, as evidenced by the significant decrease in survival rate observed in *oscst1* knockout mutant following cold stress treatment compared to WT and restored cold stress tolerance in complemented plants. Furthermore, we propose a potential molecular mechanism of *SiCST1*, suggesting its involvement in regulating key molecular processes during cold stress.

To bridge this discovery toward crop improvement, we propose two translational strategies: genetic engineering and marker-assisted selection (MAS) breeding (Xiaodi et al., 2023; Zhang et al., 2024; Wang et al., 2025b). Specifically: (1) We will construct ubiquitin promoter-driven *SiCST1* overexpression lines in rice and foxtail millet to enhance cold tolerance; (2) Through haplotype analysis of diverse germplasm, we will develop *SiCST1*-linked molecular markers for efficient identification of cold-tolerant varieties via MAS. Thus, *SiCST1* will serve as both a biotechnological target and a molecular breeding anchor bridging mechanistic insights in cold stress tolerance with practical crop resilience enhancement.

### 4.2 Interaction between SiCST1 and SiOFP1: implication for BR signaling

*SiCST1* encodes a plant-specific protein harboring a conserved RNase-H-like domain. Proteins within the RNase-H-like superfamily (RNHLS), categorized as exonucleases or endonucleases, function in critical nucleic acid metabolism processes including DNA replication or repair, homologous recombination, and RNA interference (Majorek et al., 2014). Intriguingly, recent studies identified *Reduced height 8* (*Rht8*), a key “Green Revolution” gene in wheat, as encoding an RNHLS protein that modulated plant architecture through gibberellin biosynthesis regulation (Lingling et al., 2022) (Hongchun et al., 2022). Notably, orthologs of *Rht8* in *Arabidopsis* and maize exhibited conserved roles in height determination (Lingling et al., 2022). In our study, the identification of SiOFP1 as an interactor of SiCST1 through Y2H screening provided critical insights into the molecular mechanism underlying SiCST1-mediated cold stress tolerance. Recent studies have implicated OFPs in various aspects of plant growth, development, as well as response to biotic and abiotic stresses (Wang et al., 2007, 2011; An et al., 2024). The OFPs were implicated in BR signaling pathways, which are known to play pivotal roles in plant growth, development, and stress response (Xiao et al., 2020; Cheng et al., 2023; Wang et al., 2025a; Zhou et al., 2025). Notably, several OFPs have been demonstrated to interact with components of the BR signaling pathways, such as DLT and BES1/BZR1 transcription factors (Yang et al., 2016, 2018; Xiao et al., 2020). These OFPs act as negative regulators of BR response by inhibiting these BR-signaling transcriptional complexes (Yang et al., 2016, 2018; Xiao et al., 2020). These studies suggest that OFPs may function as regulator of BR signaling, modulating the downstream response to BR. We propose that SiCST1 indirectly participates in BR signaling pathways by interacting with SiOFP1.

### 4.3 The role of CST1, OFP1 and BR signaling in cold stress tolerance

**Figure 7:**
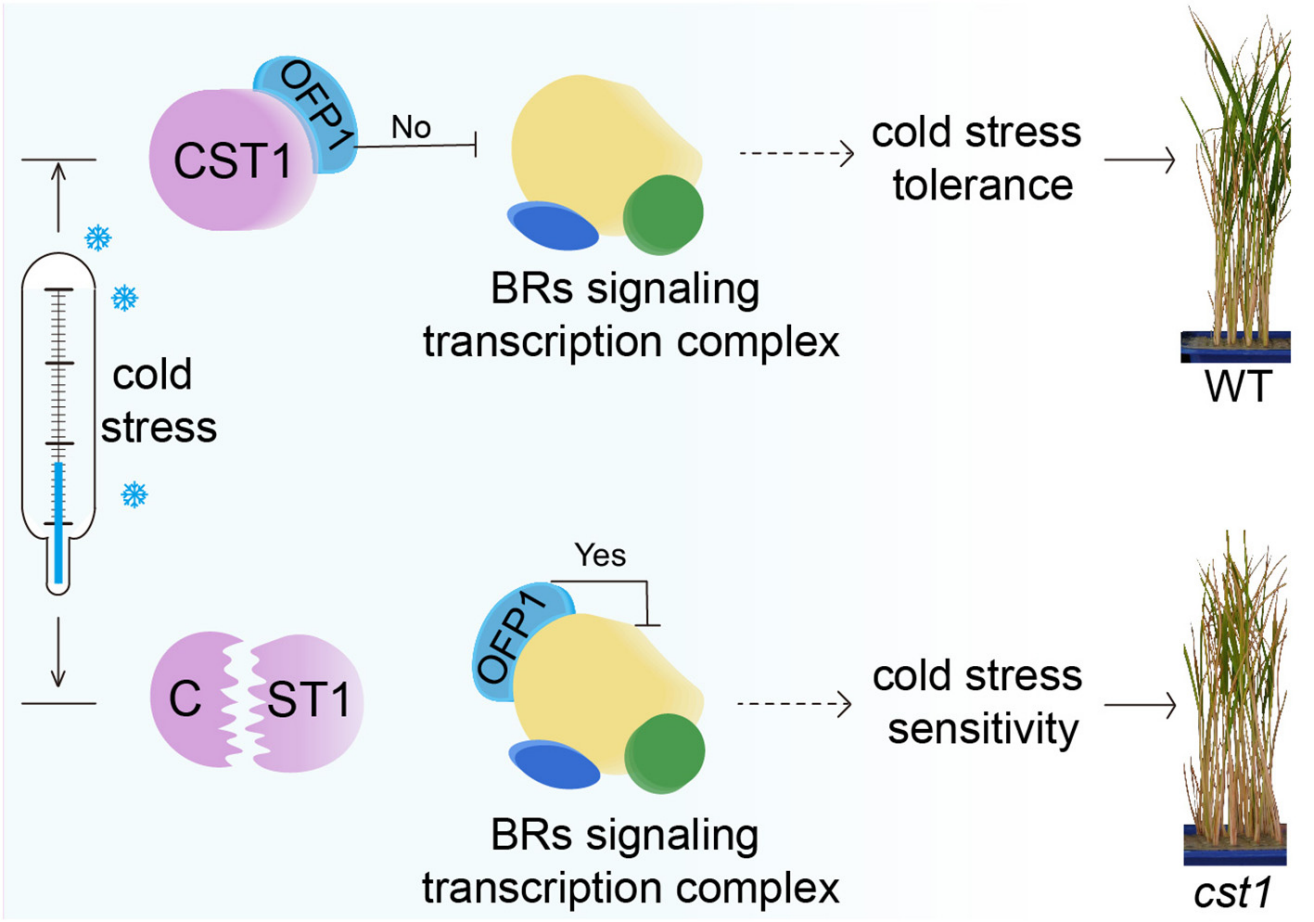
The model of CST1-OFP1 interaction coordinating BR signaling and cold stress tolerance.

Accumulating evidence suggests that BR signaling pathways were involved in the regulation of cold stress tolerance in plants (He et al., 2024). BR treatment has been shown to enhance the expression of cold-responsive genes and improve plant cold stress tolerance (Kagale et al., 2007; Anwar et al., 2018; Xia et al., 2018). Conversely, mutants defective in BR signaling exhibit reduced cold stress tolerance (Planas-Riverola et al., 2019; Chaudhuri et al., 2022). These findings collectively indicate that BR signaling plays a positive role in conferring cold stress tolerance. In our study, we speculate that SiCST1 may regulate cold stress tolerance by interacting with SiOFP1 and modulating BR signaling. The model is as follows: In WT plants, CST1 interacts with OFP1, to release its inhibition of BR signaling transcription complex, thereby activating BR signaling pathways and conferring cold stress tolerance. On the other hand, in mutant, the mutated CST1 fails to interact with OFP1, allowing OFP1 to maintain its inhibition of BR signaling transcription complex, which renders the mutant plants sensitive to cold stress (**Figure 7**). Future studies aimed at elucidating the precise molecular mechanisms underlying the interaction between SiCST1 and SiOFP1, as well as their roles in BR signaling and cold stress tolerance, will be crucial for advancing our understanding of this important regulatory pathway.

## Data Availability

The raw data supporting the conclusions of this article will be made available by the authors, without undue reservation.
