# Supplementary material for: SiCST1, a novel plant-specific protein of foxtail millet, confers cold stress tolerance in plants
Source: Front Plant Sci. 2025 Aug 5;16:1618053. doi: 10.3389/fpls.2025.1618053 (PMC12361253; doi:10.3389/fpls.2025.1618053)
Supplement: Supplementary file 1 [file Table1.docx]

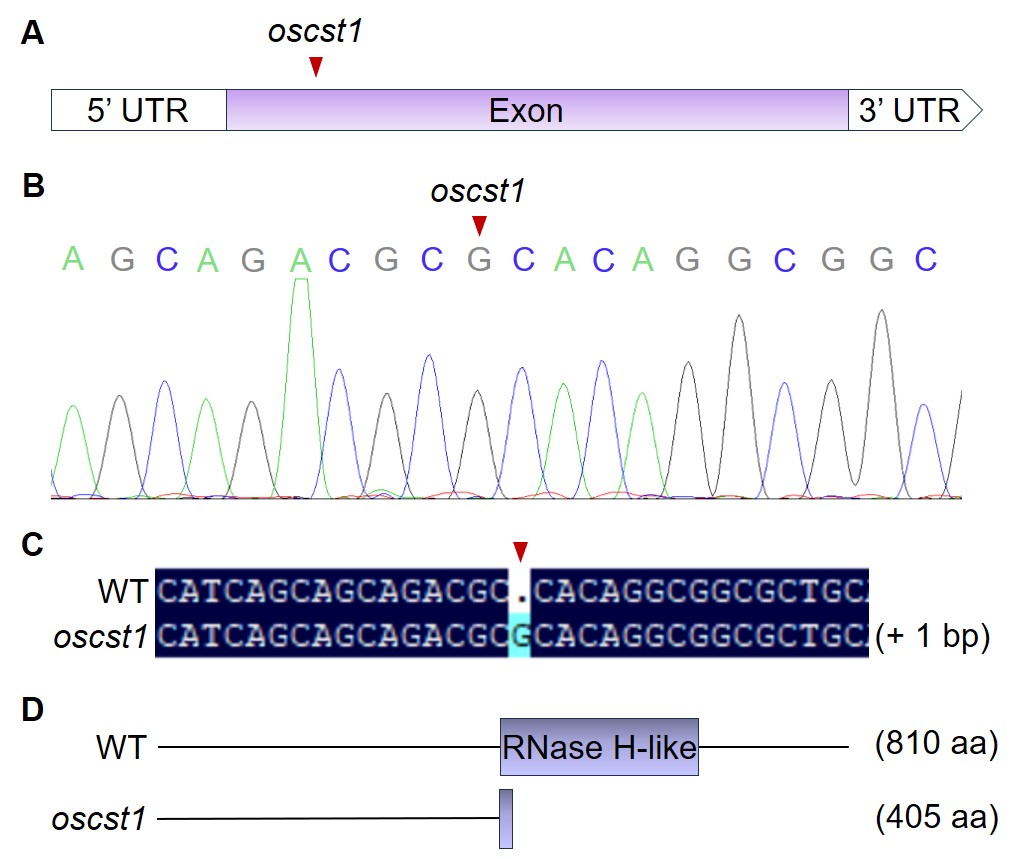
**Supplementary Figure 1.** CRISPR/Cas9-mediated gene editing targeting the single exon of *OsCST1* in rice.

(A) *OsCST1* gene structure and CRISPR target site. (B) Sequence peaks of PCR products from *oscst1*. (C) Sequencing confirmed a 1-bp insertion in the single exon of *OsCST1*. (D) The truncated protein of *oscst1* consisting of 405 amino acids lacks the C-terminal domain.


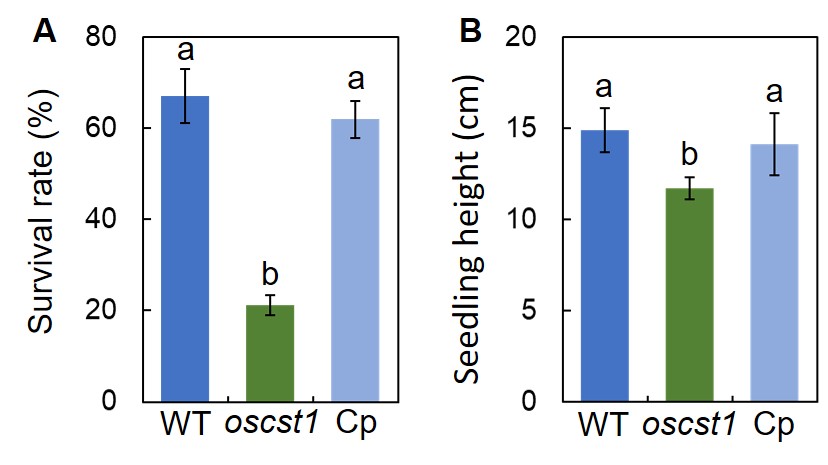


**Supplementary Figure 2.** Phenotypic comparison of WT, *oscst1*, and Cp.

(A) Survival rate was assessed following cold stress treatment at 4℃ for 96 h and subsequent recovery at 30℃ for 7 days. (B) Seedling height at three-leaf stage. WT, wild-type; Cp, complementary line. Different letters above columns indicated statistically significant differences (*LSD* test, *P* < 0.05).


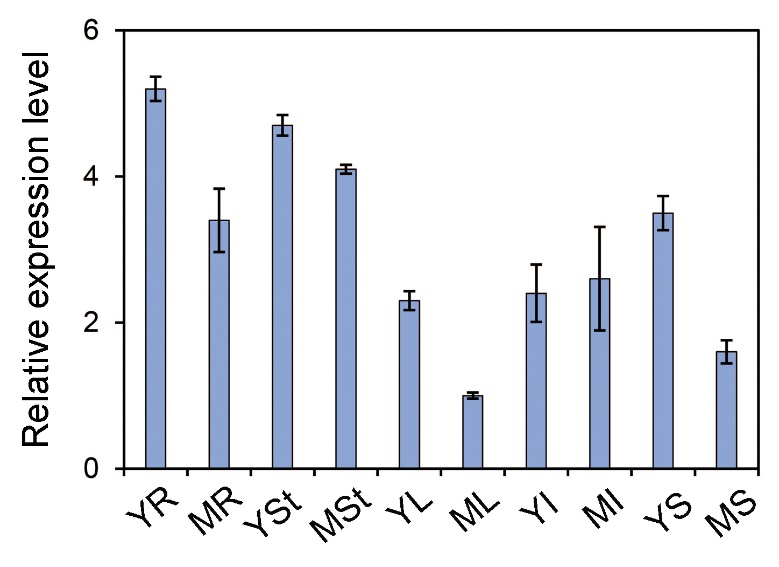
**Supplementary Figure 3.** Expression pattern of SiOFP1.

Total RNA was extracted from young roots (YR), mature roots (MR), young stems (YSt), mature stems (MSt), young leaves (YL), mature leaves (ML), young inflorescences (YI), mature inflorescences (MI), young seeds (YS), and mature seeds (MS), respectively. Values represented means ± SE (n = 15).
